# A Hygroscopic Sensor Electrode for Fast Stabilized Non-Contact ECG Signal Acquisition

**DOI:** 10.3390/s150819237

**Published:** 2015-08-05

**Authors:** Ee-May Fong, Wan-Young Chung

**Affiliations:** Department of Electronic Engineering, Pukyong National University, Busan 608-737, Korea; E-Mail: eemay88@hotmail.com

**Keywords:** capacitive electrocardiography (cECG), relative humidity, sensor electrodes, signal-to-noise ratio

## Abstract

A capacitive electrocardiography (cECG) technique using a non-invasive ECG measuring technology that does not require direct contact between the sensor and the skin has attracted much interest. The system encounters several challenges when the sensor electrode and subject’s skin are weakly coupled. Because there is no direct physical contact between the subject and any grounding point, there is no discharge path for the built-up electrostatic charge. Subsequently, the electrostatic charge build-up can temporarily contaminate the ECG signal from being clearly visible; a stabilization period (3–15 min) is required for the measurement of a clean, stable ECG signal at low humidity levels (below 55% relative humidity). Therefore, to obtain a clear ECG signal without noise and to reduce the ECG signal stabilization time to within 2 min in a dry ambient environment, we have developed a fabric electrode with embedded polymer (FEEP). The designed hygroscopic FEEP has an embedded superabsorbent polymer layer. The principle of FEEP as a conductive electrode is to provide humidity to the capacitive coupling to ensure strong coupling and to allow for the measurement of a stable, clear biomedical signal. The evaluation results show that hygroscopic FEEP is capable of rapidly measuring high-accuracy ECG signals with a higher SNR ratio.

## 1. Introduction

In recent years, coronary heart disease has become the leading cause of death worldwide. Cardiovascular diseases affect not only stressed, overweight middle-aged men in developed countries, but also women and children in low-middle-income countries. According to the 2012 World Health Statistics report by the WHO, the largest proportion of non-communicable disease deaths is caused by cardiovascular diseases (48%). It is projected that the annual number of deaths due to cardiovascular disease will increase from 17 million in year 2008 to 25 million in 2030 [1]. Therefore, it is essential to continuously monitor cardiovascular function to allow for the early detection and treatment of cardiovascular anomalies, especially in high-risk patients.

An electrocardiogram (ECG) measures the electric current generated by the heart muscle during a heartbeat. Thus, it provides useful diagnostic information about the cardiovascular system and is a strong indicator of several specific physiological and pathological conditions in humans. Conventionally, Ag/AgCl electrodes employ a conductive adhesive to ensure good contact between the electrode and subject’s bare skin, which causes skin irritation and discomfort. Therefore, a noncontact capacitive coupled ECG monitoring technique has been proposed for long-term daily health monitoring [2]. The principal goal of non-invasive measuring methods is to monitor health-related information reliably without interrupting the subject’s daily life.

Noncontact ECG monitoring has the distinct advantage of not requiring direct contact; it can measure biomedical signals through a layer of insulator such as clothes. This property allows for the integration of the measurement system into everyday objects and makes continuous measurement possible without placing constraints on the patients. Since its introduction by Lopez and Richardson [2], the applications of capacitive coupled ECG monitoring methods have been extended to various environments. For instance, Lim *et al.*, Steffen *et al.* and Aleksandrowicz *et al.* developed an unconstrained ECG measurement system built into an office chair [3,4,5] and a bed [6]; Park *et al.* applied ECG sensors on clothing [7]; Fuhrhop *et al.* integrated flexible electrodes into a belt [8]; Kato *et al.* demonstrated electrocardiography of neonates and infants using an incubator mattress system [9]; Leonhardt *et al.* proposed noncontact ECG monitoring in automobiles [10];

Despite these numerous advances, ECG signals remain very susceptible to noise from a variety of sources such as motion artifacts, electromagnetic noise, power line noise, and electrostatic charge noise. In non-contact capacitive coupled ECG measurement methods, there is no skin contact; thus, no direct connection can be made between the subject’s body and the sensor electrode. The subject’s clothing acts as an insulator between the body and sensor electrode. Occasionally, static charge builds up on the subject’s clothing. Because there is no direct physical contact between the subject and any grounding point, there is no discharge path for any static build-up; therefore, the ECG signal quality deteriorates and the signal-to-noise ratio (SNR) decreases. Furthermore, a long settling time is required to obtain a stable ECG signal.

One of the possible solutions of this problem is with advances in the design of the electrodes. Wartzek *et al.* [11] tried to design grid electrodes to overcome the noise coming from the electrostatic (triboelectric effect), however the P and T waves are not distinguishable from their acquired ECG signals. Researchers in [12,13,14] also tried to build soft dry electrodes to improve hairy skin contact, yet this method still needs direct contact with the skin. Only a few researchers in [15,16,17] have very recently called attention to the moisturization of the electrodes. In this study, we developed a fabric electrode with embedded polymer (FEEP) that takes into account the relative humidity of the environment and sensor electrode characteristics to remove the static charge rapidly and obtain a clear ECG signal. The designed FEEP with sandwiched layers ensures a high relative humidity because less static charge builds up and electrostatic charge discharges quickly. Our results show that a good quality ECG signal can be recorded with good SNR, making this system a very promising non-contact biomedical signal acquisition technology for use in ubiquitous home healthcare applications.

## 2. System Design

**Figure 1:**
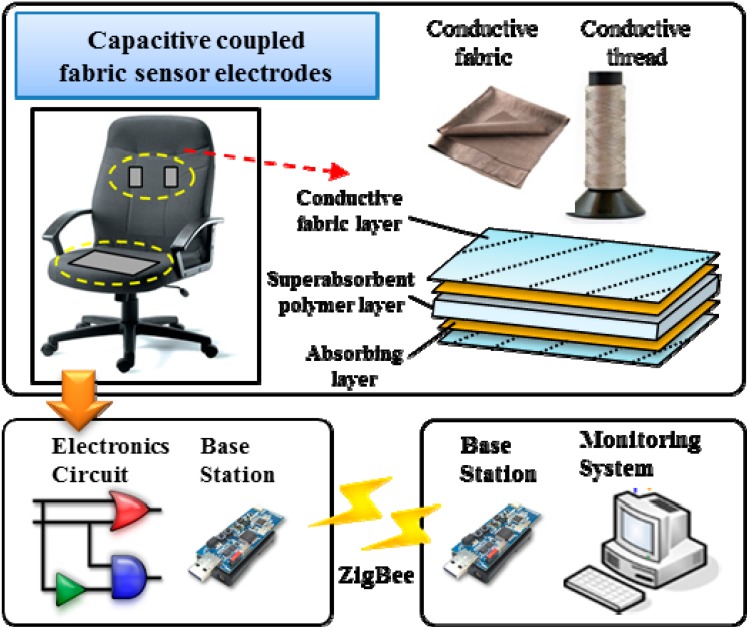
Non-contact ECG monitoring system.

The overall system architecture for our non-contact healthcare monitoring system, shown in Figure 1, comprises a pair of capacitive electrodes, a piece of conductive textile, an electronic circuit, and wireless modules to transmit and receive biomedical data wirelessly via IEEE 802.15.4. The biomedical signals transmitted from the wireless sensor node are saved and analyzed for healthcare monitoring purposes [18].

The capacitive coupled electrodes carry an alternating bioelectric current through the capacitive coupling formed by the conductive electrode, an insulator, and the skin of the subject [2]. This combination forms a capacitive coupling between the subject’s body and the sensor electrode. Our method is operational through a pair of capacitive coupled electrodes installed on the chair back and a conductive textile installed on the seat for capacitive driven-right-leg (DRL) grounding. A DRL circuit [19] has been designed as an additional reference to suppress the interference caused by the finite common-mode rejection ratio (CMRR) of the instrumental amplifiers. In the system, a hygroscopic capacitive coupled fabric sensor electrode is designed as a multiple layer sandwich structure using conductive fabric.

The capacitive electrodes are connected to an electronic circuit including high input impedance amplifiers and band-pass filters for analog signal processing. These include a low-pass filter (100 Hz), high-pass filter (0.04 Hz), and notch filter (60 Hz). In addition, the system included a sensor node with an integrated ultra-low-power microcontroller. The MSP430 digitizes the ECG signals with its built-in 12-bit analog-to-digital converter and the CC2420 wireless transceiver in the wireless sensor node transmits the biomedical signal data through via 802.15.4 using a ZigBee-based radio protocol (frequency band: 2.4 GHz to 2.485 GHz) at a transceiver rate of 250 Kbps to a PC-based monitoring system.

## 3. Sensor Materials and Performance Analysis

### 3.1. Problem Statement

**Figure 2:**
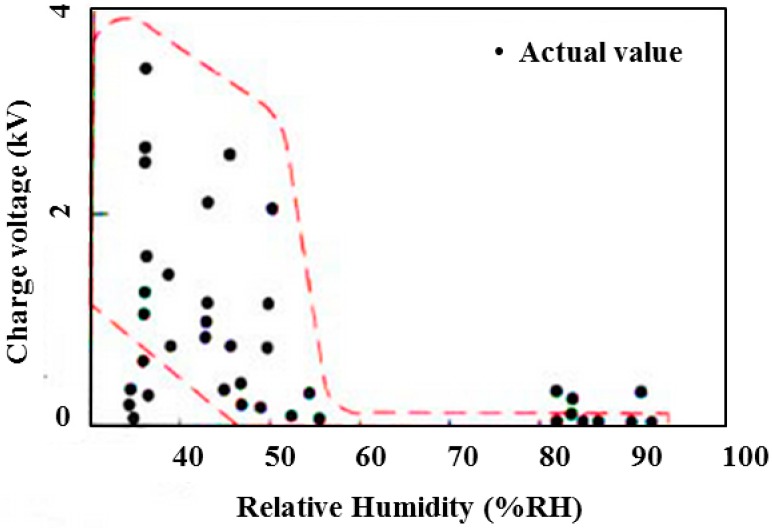
Relationship between humidity and static electricity (with permission from [21]).

In non-contact ECG measurement approaches, the capacitive coupling is formed between the body and the sensor electrode. To achieve a high capacitance value and stable coupling of the capacitive electrodes, an insulator such as a cotton shirt (which has a high dielectric constant) was used in the test. However, electrostatic charge on the shirt prevents the acquisition of a clear cardiac signal. It has been proven that the triboelectric effect is dependent upon ambient humidity [20], therefore, electrostatic charge often builds up on the subject’s clothing at low relative humidity, especially at less than 55% relative humidity [21]. Figure 2 illustrates the relationship between environmental humidity and static electricity.

Thus, when the environmental humidity is low, a large electrostatic charge accumulates on the insulator, and because there is no direct physical contact between the subject and any grounding point, there is no path for the electrostatic charge to be discharged. Thus, the buildup of electrostatic charge on the clothing causes two main problems in noncontact measurement systems.

First, it significantly affects the coupling capacitance. The humidity level induces an electrostatic charge, and it affects the dielectric constant of air and the insulating material (cotton shirt). As humidity increases the dielectric increases [22]. Generally, the permittivity is not a constant; it can depend on the strength of the electric field. The permittivity (ε_r_) of atmospheric air and other insulating materials are functions of moisture content [23]. Thus, this results in poor signal quality and a low SNR. Consequently, inaccurate acquisition of health data and erroneous decision making may occur.

Second, the buildup of electrostatic charge leads to a long stabilization time for clear, stable signal measurement, therefore static discharge is required under changing humidity of clothes to ensure a clear signal measurement, and to reduce stabilization time.

Previous studies [24,25] have addressed this problem but no solutions have yet been presented. Leonhardt *et al.* found that an ECG signal will only remain stable after approximately 250 s in their system [10]. Wartzek *et al.* [11] analyzed the triboelectric effects in capacitive biopotential measurement and proposed an actively driven grid for a fast discharge of electrostatic charge. Lowne [26] noted the main issues of non-contact ECG monitoring systems, such as electrostatic charge build- up and long settling times.

### 3.2. Sensor Electrode Types

To resolve the previously discussed issues, the characteristics of different kinds of electrodes were evaluated, as shown in Table 1. The relationship between humidity and electrostatic charge was also analyzed. According to the statistical chart [20], static charge can be avoided when the relative humidity is maintained at a value higher than 55%–60%. Thus, problems arise when the relative humidity is lower than 55%–60%. The average relative humidity in spring and winter in many Asian, North American and European countries is between 40% and 50%. During these seasons, the practical application of cECG methods can face significant obstacles. During the monsoon season, however, the relative humidity is typically above 70%, and static charge is not problematic.

**Table 1:** Characteristic and specification of various sensor electrodes.

	PCB Electrode Plate (PEP)	Conductive Fabric Electrode (CFE)	Fabric Electrode + Polymer (FEEP)
	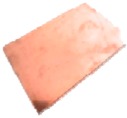	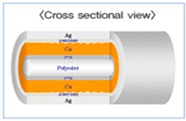	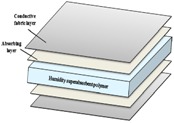
*Mx ^a^*	Copper coating	Polyester with coating Cu and silver	Polyester fabric electrode + superabsorbent polymer
*Wg*	128 ± 5	83 ± 5	173 ± 5
*T*	0.8 ± 0. 01	0.1 ± 0.01	1.5 ± 0.01
*Bs*	798 ± 10	661.3 ± 10	661.3 ± 10
*Ω*	less than 0.01	less than 0.04	less than 0.04
*Se*	85	93	93
*Er*	No	No	Yes

^a^ Symbol represents the characteristics of various sensor electrodes; *Mx* = material, *Wg* = weight (g/m^2^), *T* = thickness (mm); *Bs* = breaking strength (N), Ω = surface resistance, *Se* = shielding effectiveness (dB), *Er* = Efficiency at low RH.

A PCB electrode plate (PEP), a conductive fabric electrode (CFE), and a conductive fabric electrode with embedded polymer (FEEP) are used as sensor materials. With the aim of reducing the accumulated charge and decreasing the discharge time, we developed a conductive fabric electrode with an embedded superabsorbent polymer as sensor material to rapidly discharge any accumulated charge.

**Figure 3:**
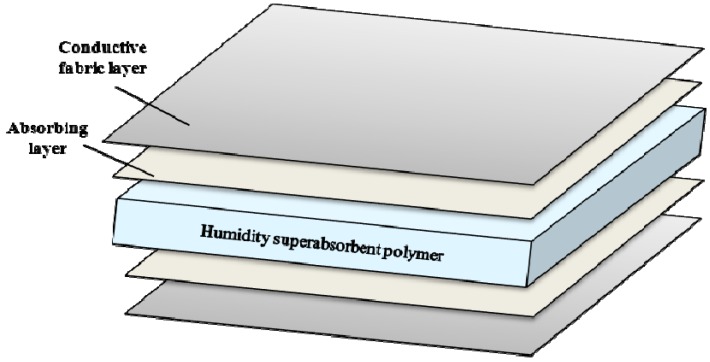
Fabric electrode with embedded superabsorbent polymer.

The proposed fabric electrode is shown in Figure 3; the FEEP is built with an integrated superabsorbent polymer (H-600 polymer) [27] to provide humidity. In the FEEP, the superabsorbent polymer acts as a sponge humidifier embedded in the fabric electrode. The merit of superabsorbent polymer is that they instantaneously and rapidly absorb any aqueous solution on contact, hold large quantities of fluid, and emit moisture into the air when the environmental humidity is low, helping to regulate humidity [28]. Therefore, when the environmental relative humidity is higher, less static charge is built up on the capacitive coupling; thus, a shorter discharge time is achieved, and a clearer, more stable signal can be measured.

The area of the fabric electrode is designed to be 4 cm × 4 cm. This sensor is designed around the embedded superabsorbent polymer in the middle of the electrode. Two absorbent cotton layers are used to maintain the humidity of the polymer on both the top and bottom of the sensor electrode. The conductive fabric forms the outer layer of the fabric electrode for sensing. An additional advantage of this fabric electrode is that it is flexible; the fabric electrode can bend according to the curvature of the subject’s body.

### 3.3. Signal-to-Noise Ratio

To evaluate the sensor performance in long-term monitoring, noncontact biomedical signal acquisition is carried out continuously for 20 min. Experiments are carried out with different types of sensor electrodes. The measurement starts at ambient room temperature of 25 °C and relative humidity of around 50%. The mean of both magnitudes of QRS complexes and SNRs at several intervals are measured and recorded. The SNR (dB) can be calculated as:

(1) $$SNR_{dB}=20\;\log_{10}\left(\frac{A_{signal}}{A_{noise}}\right)$$

where *A_signal_* is the amplitude of a measured ECG signal, *A_noise_* is the noise signal which is obtained from the measured ECG signal.

## 4. Experimental Results

All experiments were carried out in a laboratory. The temperature was kept constant at approximately 25 °C with a relative humidity of approximately 50%. Subjects wore a cotton t-shirt and jeans. A PEP, a CFE, and a conductive FEEP were used as sensor materials. The electrode size was designed to be 4 cm × 4 cm and the total thickness of the sensor electrode was approximately 20 mm. They were fixed with a vertical center-to-center distance of 10 cm. The sensor electrodes were attached at a fixed position on the chair. For the driven ground plane, a conductive textile with a size of 30 cm × 30 cm was employed.

### 4.1. Performance of Various Electrodes

**Figure 4:**
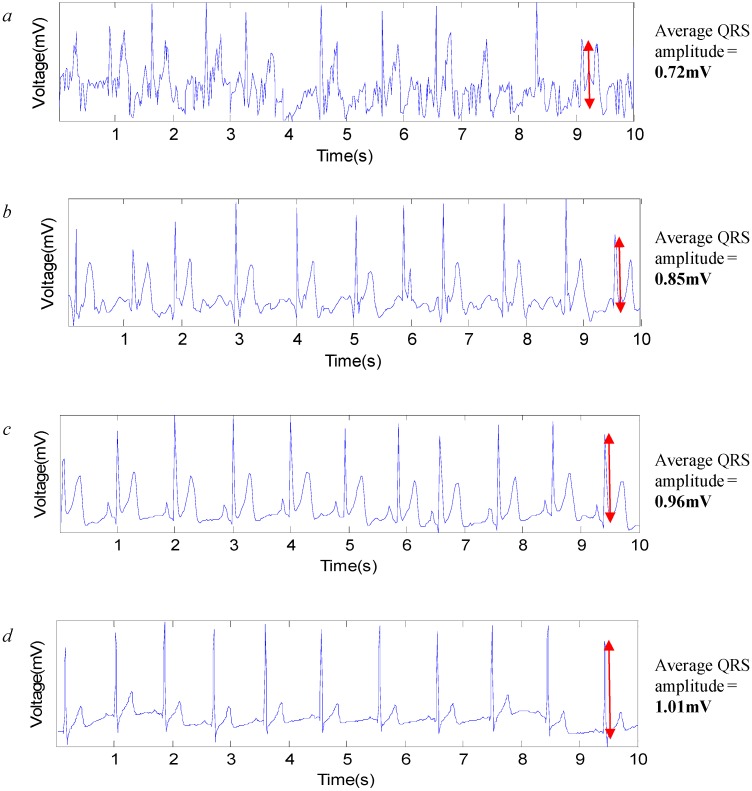
ECG signals captured with different sensor electrodes at different times. (**a**) CFE electrode at the first minute; (**b**) CFE electrode at the tenth minute; (**c**) FEEP electrode at the first minute; (**d**) FEEP electrode at the tenth minute.

Different electrodes are investigated to analyze their feasibility for use in long term monitoring. Figure 4a,b shows the ECG signals recorded at different timings with the CFE; Figure 4c,d presents the ECG signals captured with the FEEP.

The results show that with the CFE and FEEP, QRS complexes are visible in the signal, yet the signal quality varies significantly over the recording timing. For both electrodes, some stabilization time is required for a strong coupling between the electrode and the body due to the variation in impedance, changes in moisture, and discharge of static charges. Therefore, strong interference signals due to the static charge effect appear at the very beginning of the experiments (in the first minute) as shown in Figure 4a,c. The performance improves after some stabilization time, such as during the tenth minute, as shown in Figure 4b,d.

For ECG signals measured with the CFE and FEEP, we observed that the FEEP produced a clearer ECG signal with a higher QRS amplitude (as shown in Figure 4c,d) as compared to the CFE (as shown in Figure 4a,b). This difference can be attributed to the FEEP ensuring high relative humidity with its integrated superabsorbent polymer. At high relative humidity, the resistance of most dielectrics is reduced, which results in an increase in return current, which is the current that opposes static charge buildup. Hence, the FEEP reduces the accumulated charge and decreases discharge time by the increment of moisture levels between the body and the sensor electrode, and ensuring that clear, stable ECG signal can be measured.

### 4.2. Performance Analysis

**Figure 5:**
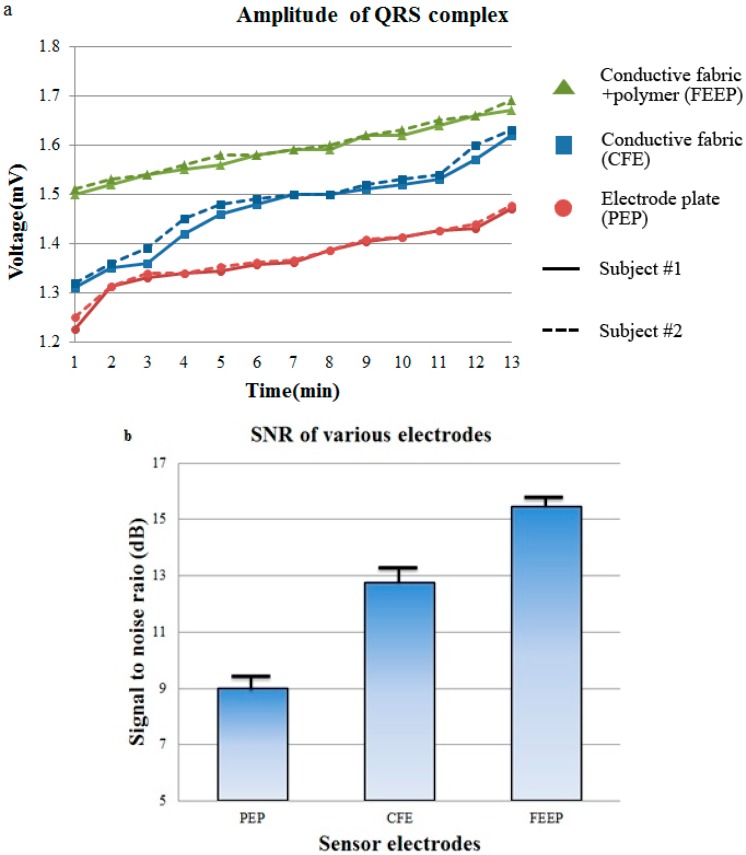
Performance analysis of the monitoring system. (**a**) Amplitudes of QRS complexes for each sensor electrodes tested on two subjects; (**b**) Means and standard deviations of the SNRs for each sensor electrodes.

We took two subjects’ performances as representatives for the comparison of QRS complex from PEP, CFE and FEEP electrode pairs. The capacitive ECG signal is very sensitive to the location of the electrode pairs. Thus, three electrode pairs were located at the same location when we measured the ECG signals. This means that the person has to stand up and sit down again before the next electrode material is used. We measured the QRS complexes every 2 min for each sensor electrode and 10 times for each subject, and then calculate the mean value. Each electrode is tested on two males. Then, the mean SNRs of the representative two males are calculated, as shown in Figure 5. Meanwhile, we measured the relative humidity using a humidity sensor on the sensor node to check the relationship between them. The experiment starts at room temperature (25 °C); the starting relative humidity is approximately 50% for the CFE and FEEP cases. At the fifth minute of the experiment, the CFE experiences 55% relative humidity, whereas the FEEP experiences 75% relative humidity at the location of the conductive electrode surface. At the tenth minute of the experiment, the CFE experiences 60% relative humidity whereas the FEEP experiences 85% relative humidity at the location of the conductive electrode surface. The increase of the relative humidity at the location of conductive electrodes surface is assumed due to the fact that the moisture from the superabsorbent polymer of the FEEP increases the relative humidity at the electrode surface after the subject is sitting on the chair. The moisture at the superabsorbent of FEEP could be absorbed from body sweat or ambient humidity.

Figure 5a indicates the results for two subjects on three different sensor electrodes. At the beginning of the experiment, the PEP measures a small amplitude QRS complex (at 1.22 mV) and the CFE measures an amplitude QRS complex (at 1.31 mV), whereas the proposed FEEP measures a higher amplitude QRS complex (at 1.50 mV) for subject #1. Again, this effect is caused by the weak coupling and induced static charge. At the tenth minute of the experiment, the PEP measures a higher QRS amplitude (approximately 1.41 mV) and the CFE measures a QRS amplitude (approximately 1.53 mV), whereas the FEEP measures a QRS complex of 1.62 mV. For the CFE, the performance improves gradually because of the increased humidity from the subject’s body and it takes a long time (approximately 10 min) for the amplitudes of the QRS complexes to increase gradually such that a clearer ECG signal can be observed. However, with the proposed FEEP electrode, a higher QRS amplitude and clearer signal can be seen more quickly for both subjects; and we have proven that the FEEP electrode acts as a high-performance sensor electrode in an optimal system.

As can be seen in Figure 5b, the PEP has the lowest SNR at approximately 9.0 dB and whereas the CFE has an SNR of 12.8 dB and the FEEP has an SNR of 15.5 dB, which is poor performance for a non-contact ECG monitoring system. ECG signals are masked by noises and the QRS amplitudes are barely visible. The ECG signal obtained by the dry CFE is better than the signal by the dry PEP. The better performance of dry CFE than the dry PEP could be due to the bendable material which adapts to the body shape and enhances signal coupling, as explained in [13]. However, the FEEP achieves good performance with a large QRS amplitude and good SNR of approximately 15.5 dB. This improvement can be explained by the strong coupling, built up rapidly under higher relative humidity when the FEEP is employed. Thus, static charge dissipates rapidly in materials with high relative humidity, which results in a good SNR.

### 4.3. Comparison with Other Sensor Electrodes

The non-contact capacitive biopotential measurement technique has been widely used. At the same time, non-contact electrodes with the objective to improve electrode-body interface have been presented by many researchers. This is important to eliminate triboelectricity effects due to friction and static charge, and to suppress motion artifacts to minimal values. Table 2 provides an overview of existing sensor electrodes developed by researchers. Del Re *et al.* [29] enhanced a bio-electrode with a a cushioning layer placed between the body and electrode whereby this enhancement layer can carry and release water or moisture to reduce triboelectric noise. Chi *et al.* [30] mentioned the poor settling times but the solution provided distorted the signal waveform. Grützmann *et al.* [12] presented a soft dry electrode where a soft foam cushioning layer is constructed on top of a SiO_2_ electrode to improve the contact on hairy skin to diminish motion artifacts. A passive filter network is also integrated into the new electrodes to suppress the slow offset fluctuation of the ECG signal. Wartzek *et al.* [11] also described the triboelectricity effects. Four different types of sensor electrodes were constructed to analyze the electrostatic voltage generated and the discharging behavior. Wartzek *et al.* proposed an insulated electrode, metal-coated electrode, no-isolation electrode and grid electrode to reduce triboelectricity charges. Experimental analysis proved that a metal layer provides a conductive back-path for separated charges and the grid on top of the electrode produces much less electrostatic voltages as charges are separated on the material’s surface, so the grid can drain them off. The discharge behaviors of different electrodes are different. Leicht *et al.* [15] and Weder *et al.* [16] used superabsorber or absorbent layers to moisturize the electrodes to reduce the motion artifact noise and to get long time signal stabilization in recent work.

**Figure 6:**
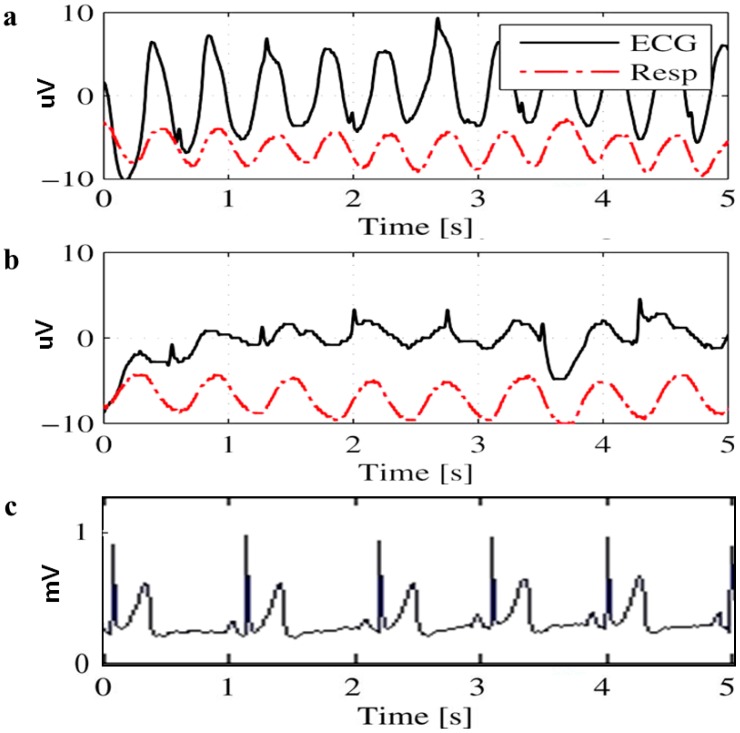
Comparison of ECG signals measured from grid-electrode (with permission from [11]) and the proposed FEEP. (**a**) The active driven grid is switched off (it acts as metal-coated electrodes); (**b**) The active driven grid is switched on; (**c**) The proposed sensor electrodes FEEP.

However in [11,25], the researchers showed a fluctuating ECG signal as shown in Figure 6. Figure 6a shows the QRS complexes are rarely visible when the grid is turned off. Meanwhile, even the grid electrode produces much less electrostatic voltage and the discharge slope is much larger (can quickly drain off the charge and discharge the cloth), and the ECG signals measured with a grid electrode do not show a clear ECG signal with clear P peaks, and T peaks. QRS complexes with small ampliyude are visible, as shown in Figure 6b. From a medical field point of view, the P waves and T waves are important for the diagnosis of diseases such as arrhythmia, Wolff-Parkinson-White syndrome, myocardial ischemia and infarction. As compared to ECG signals measured with the proposed FEEP electrodes, P waves, QRS complexes, T waves are clearly distinguishable which are very useful for cardiological diagnostics during the interpretation of abnormal ECGs. Figure 6c shows the ECG signals measured with the proposed sensor electrode FEEP where the P waves, QRS complexes, T waves are clearly visible. Secondly, the authors in [23] mentioned that if charges are separated between fibers, the grid does not improve the results as it does not reduce electrostatic charge.

Meanwhile, some researchers [14] developed a sensor electrode with on-board electronics components. From a long term point of view, this may result in a high cost sensor electrode replacement when the electronics component or op-amp is not functioning. Users have to replace the sensor electrodes with on-board components. The proposed FEEP electrode however can be replaced easily at very low cost. Besides, FEEP sensor electrodes are easy to build using conductive fabric and superabsorbent polymers. They are also very flexible and it can bend according to the body curvature and therefore, very minimal movement artifacts occur once the coupling capacitance is formed.

**Table 2:** Overview of existing sensor electrodes.

Author [Ref.]	Problem Statement	Solutions	Features
Brun del Re [29]	Triboelectric noise with orders of magnitude larger than the desired body signal arises when obtain signal.	A cushioning layer is placed intermediate the body and electrode where this enhancement layer to carry and release water or moisture To reduce triboelectric noise.	On-board electronic components on electrode will make electrode replacement expensive.
Chi [30]	Poor settling times due to the high-pass characteristic at the electrode. Recovery times of upwards of 10 s.	Shifting the corner frequency of the high-pass filter to improve settling time, but at a cost of distorting the signal waveform.	-
Wartzek [11]	Global triboelectricity – Common-mode interference on the whole body Local triboelectricity – Electrode-body interface.	Electrode design Insulated electrode Metal-coated electrode No-isolation electrode Grid electrode.	P peaks and T peaks of ECG signal are not distinguishable.
Gruetzmann [12]	Motion artifacts such as walking, breathing introduce fluctuations in the zero line.	Soft dry electrode to improve the contact on hairy skin to reduce the electrode impedance, to diminish motion artifacts. Passive filter network to suppress slow offset fluctuation of the ECG signal.	Soft dry electrodes are developed to improve hairy skin contact, which is not applicable for non-contact measurement technique.
Chi [14]	Dry electrodes are prone to poor signal quality due to unstable offsets, high drifts, long settling times, movement artifacts.	Conductive media layer To aid in conduction due to its high ionic content. Ionic exchange media To protect conductive media from damage To help retain its moisture content.	Dry electrodes are built for direct contact ECG measurements.
Leicht [15]	Strong artifact noise is generated at capacitive electrodes for a car seat due to driver movement	Superabsorber to moisturize the electrodes to reduce strong artifacts and triboelectricity	Superabsorber layer integrated in the electrode generates moisture to reduce motion artifacts and triboelectricity.
Weder [16]	Water vapor has a positive effect on ECG quality in a breast belt for the long time monitoring of ECG	Absorbent layer to keep condensed sweat water at flexible water tank in a form of absorbent layer to moisturize electrodes with a very low amount of water vapor	Electrodes are moisturized with a very low amount of water vapor from the integrated reservoir
The proposed electrode FEEP	Long stabilization time is needed to allow for static discharge.	Sandwiched layer hygroscopic FEEPConductive fabric electrode with embedded superabsorbent polymers (acts as sponge humidifiers) To shorten stabilization time. To rapidly discharge any accumulated charge.	Low cost Flexibility Easy to build Sensor electrode can be replaced easily

## 5. Conclusions

Using the capacitively coupled ECG measurement system presented here, ECG signals can be obtained without direct skin contact, and consequently without causing skin irritation. By integrating these technologies into a chair system in a home healthcare environment, biomedical data can be acquired through clothing in a nonintrusive fashion. By adapting a hygroscopic FEEP, high humidity conditions can be ensured. The stabilization time is shortened and a clear, stable ECG signal quality with high QRS amplitude and SNR can be obtained, even in environments with relative humidity lower than 55%–60%. Thus, problems regarding static charge and long stabilization times in ubiquitous healthcare systems can be resolved
